# Chloride Distribution and Steel Corrosion in a Concrete Bridge after Long-Term Exposure to Natural Marine Environment

**DOI:** 10.3390/ma13173900

**Published:** 2020-09-03

**Authors:** Jun Liu, Zhilu Jiang, Yulong Zhao, Hao Zhou, Xiaodong Wang, Haijun Zhou, Feng Xing, Shanglin Li, Jihua Zhu, Wei Liu

**Affiliations:** Guangdong Provincial Key Laboratory of Durability for Marine Civil Engineering, Shenzhen University, Shenzhen 518060, Guangdong, China; liujun@szu.edu.cn (J.L.); 1800331020@email.szu.edu.cn (Y.Z.); yidan.huang@innoventbio.com (H.Z.); wangxiaodong@email.szu.edu.cn (X.W.); haijun@szu.edu.cn (H.Z.); xingf@szu.edu.cn (F.X.); lishanglin@email.szu.edu.cn (S.L.); zhujh@szu.edu.cn (J.Z.); liuwei@szu.edu.cn (W.L.)

**Keywords:** steel reinforced concrete, raman spectroscopy, rust, chloride transport

## Abstract

Chloride-induced steel corrosion is the most concerning issue for the durability of concrete structures. Concrete and steel samples were obtained from a 30-year-old reinforced concrete bridge. The chloride content was measured by a potentiometric titration method and the microstructure of concrete was obtained by scanning electron microscopy and mercury intrusion porosimetry. The rust phases of the steel were detected by X-ray diffraction and Raman analysis. It was found that the convection depth for chloride transport in cracked concrete was significantly larger than that in uncracked concrete. The concrete in a pier column facing upstream had greater porosity due to the water impact and calcium leaching. The coefficients of variability of chloride diffusivity of concrete for the bridge deck and the pier column were significantly different. Rust phases including lepidocrocite, goethite, akaganeite, magnetite, and maghemite were detected using Raman spectroscopy and X-ray diffraction. The major phases of steel rust in the atmospheric zone were lepidocrocite and goethite, while they were lepidocrocite and maghemite in the tidal zone. The results of this study would provide information concerning the chloride-induced steel corrosion under a marine environment in order to predict long-term behaviors of a reinforced concrete structure.

## 1. Introduction

The degradation of reinforced concrete (RC) structures in a marine environment is related to physical and chemical processes including chloride penetration, sulfate attack, and carbonation. Among these factors, chloride penetration is the most concerning for the durability of concrete structures [1,2]. Costa and Appleton [3] studied concrete structures exposed to a marine environment in Portugal and found extensive deterioration due to chloride-induced corrosion of the reinforcement. When the chloride content at the steel surface reaches a critical level, the passive layer of the steel is destroyed and steel corrosion occurs [1,4,5,6]. Expansion of corrosion products may cause cracking and spalling of the cover concrete, which will then no longer protect the reinforcing steel effectively. Furthermore, the mechanical properties of steel bars were greatly impacted by the increase in corrosion degree [7,8].

Chloride ions move into concrete by the mechanisms of diffusion and convection. Diffusion is caused by differences in chloride ion concentrations in pore water while convection is due to water transport carrying chloride ions. During the chloride transport process, some chloride is dissolved in pore water and the rest is bound by cement hydrates, i.e., free chloride and bound chloride, respectively. The free chloride is responsible for destroying the passive layer of steel and initiating steel corrosion [9].

In a tidal zone, concrete is subject to periodic drying and wetting processes during the ebb and rise of the tide. During wetting periods, chloride ions are brought into concrete along with seawater absorption. During drying periods, chloride diffuses from the exposed surface to the deeper areas that have a lower chloride concentration. Cyclic wetting and drying conditions can significantly accelerate chloride penetration in concrete. Field tests for a concrete harbor deck in the tidal zone were carried out at different altitudes by Zhang and Jin [10]. The proportion of drying time in a wetting-drying cycle increased with increasing altitude. The results showed that chloride accumulation first increased and then decreased as the drying time increased. In addition to chloride from seawater, atmospheric chloride in the marine environment accumulates on concrete surfaces and slowly diffuses into the concrete [11]. McGee [12] investigated the surface chloride contents of 1158 bridges in the Australian state of Tasmania and found that surface chloride content was a function of distance from the coastline. Liu et al. [13] investigated the influence of water-cement ratios and fly ash on the surface chloride concentration. In the atmospheric zone, concrete carbonation can influence chloride transport by modifying pore structures and decreasing chloride binding capacity [14,15].

The steel bar embedded in concrete is initially covered by a thin passive film of iron oxides [16]. Chloride ions on the steel surface break down the passivity of the film by three mechanisms [17] (penetration, film breaking, and adsorption), which initiate corrosion when chloride ion content reaches a critical value. The determination of an indicator for the corrosion initiation becomes crucial for the prediction of reinforced concrete structures’ service life. It is widely believed that chloride content and concrete alkalinity both affect the initiation of steel corrosion. The corrosion may still occur with a low chloride content when concrete alkalinity decreases. Therefore, the parameter [Cl^−^]/[OH^−^] was used as an indicator for the corrosion initiation by researchers [18,19,20]. The threshold value for the [Cl^−^]/[OH^−^] ratio ranges from 0.12 to 3.0 [21]. The wide range of the critical value was probably due to different test methods and identifying standards for depassivation of reinforcement. Steel corrosion is an electrochemical process that includes anodic and cathodic reactions, during which chloride accelerates the reactions through depolarization effects and ion channels. The product at the anode, Fe^2+^, then reacts with hydroxide ion in the concrete pore solution, forming the transition product Fe(OH)_2_, which will be oxidized to Fe(OH)_3_. As corrosion time increases, dehydration of Fe(OH)_2_ occurs, leading to the formation of lepidocrocite (γ-FeOOH), which is the main rust phase at the initial stage of corrosion. Under a chloride-contaminated environment, another rust, akageneite (β-FeOOH), forms through unstable phase green rust, (3Fe(OH)_2_∙Fe(OH)_2_Cl∙nH_2_O). It was found that the structure of β-FeOOH was looser than γ-FeOOH, and β-FeOOH favored pitting corrosion, which would accelerate the corrosion of reinforcement [22,23]. Under certain conditions, γ-FeOOH will be further transformed to more stable rusts, such as goethite (α-FeOOH) and magnetite (Fe_3_O_4_). Corrosion products are composed of different oxides and oxyhydroxides with higher volumes than iron. Expansion due to conversion of iron to corrosion products can lead to cracking of cover concrete. The expansion can be better understood if the rust composition is known. Criado et al. [24] studied steel rust on reinforcing steel embedded in mortars containing chloride by Raman spectroscopy and found that the main corrosion products were iron with low crystallinity, α-FeOOH and γ-FeOOH. Poupard et al. [25] tested steel bars in a RC beam in a marine environment by X-ray diffraction (XRD) and μ-Raman analysis. They identified α-FeOOH, β-FeOOH, Fe_3_O_4_, and maghemite (γ-Fe_2_O_3_) as corrosion products. The formation of rust phases is dependent on both steel materials and exposure conditions.

Much laboratory work [26,27,28] has been done on chloride-induced corrosion of steel bars embedded in concrete. The advantage of a laboratory study is that environmental conditions can be specifically controlled in order to investigate the effects of different factors on the corrosion systematically. However, the mechanism of chloride-induced corrosion in the field may be different from the mechanism under accelerated conditions in laboratory. Field studies are important because they improve our understanding of corrosion processes under in-situ conditions. Several works [29,30,31,32] have been conducted to study the long-term corrosion behavior of RC structures in the field, and different corrosion mechanisms have been analyzed. Duffó et al. [29] investigated carbonation-induced corrosion of steel bars embedded in concrete slabs for more than 65 years from a building in Buenos Aires, Argentina. The corrosion potential of the rebars was measured by using a copper-copper sulfate reference electrode, and the rusts were analyzed by scanning electron microscopy (SEM), optical microscopy, XRD, and Mössbauer spectroscopy. The results indicated that the corrosion products consisted of an inner layer composed mainly of magnetite and an outer layer composed of α- and γ-iron oxyhydroxides. Gartner et al. [31] monitored corrosion of steel reinforcement in concrete columns exposed to different zones of a real marine environment for 52 months. It was found that electrical resistance probes and coupled multi-electrodes were effective methods for monitoring corrosion of concrete members in the long term. Their results also showed that stainless steel in such a corrosive environment had better performance than ordinary carbon steel. Fattah et al. [32] confirmed the effectiveness of supplementary cementitious materials and chemical agents for reducing chloride ingress under both laboratory and field conditions. Otieno et al. [33] compared chloride-induced corrosion of beam specimens exposed to accelerated laboratory conditions (cyclic wetting and drying) and a filed marine tidal environment. The corrosion rates of steel bars in the beams were measured by the coulostatic technique, during which half-cell potential was measured simultaneously. The results showed that the corrosion performance in the laboratory environment cannot be related to the performance of reinforced concrete in the field.

In the previous study on the steel corrosion embedded in concrete, most of the work has been carried out in the laboratory under accelerated conditions, due to the slow processes of corrosion under a natural environment. On the other hand, several studies which conducted field investigations revealed that the corrosion rate and compositions of corrosion products are caused by different corrosion patterns. However, the influence of different zones in the marine environment on chloride-induced corrosion has not been understood thoroughly. Chloride penetration in concrete and steel corrosion behavior have seldom been studied simultaneously in a real structure. The purpose of this study is to investigate the degradation of concrete and steel subjected to different zones in a real marine environment. The chloride profiles and steel corrosion were studied in the atmospheric and tidal zones for a 30-year-old concrete bridge in a marine environment in Shenzhen, China. Concrete microstructures were investigated by SEM and mercury intrusion porosimetry (MIP). Corrosion products of steel in the atmospheric and tidal zones were identified by XRD and Raman analysis.

## 2. Experimental Program

### 2.1. Description of the Tested Bridge

**Figure 1:**
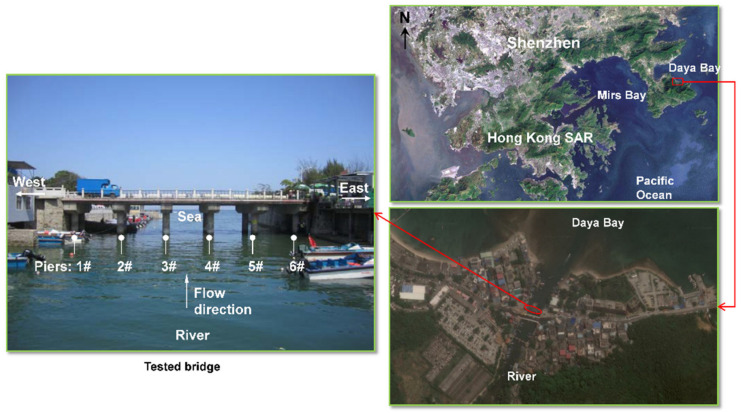
Geographic location of the tested bridge.

The aim of the research is to clarify the behaviors of chloride penetration and steel corrosion in a real marine environment. The chloride distribution in concrete, rust compositions, and morphology of embedded steel in a bridge will be tested. The tested bridge spans the entrance of a river into the Daya Bay on the Dapeng peninsula in the city of Shenzhen, China. The geographic location of the bridge is shown in Figure 1. It is a cast-in-situ RC bridge with a service time of around 30 years. The bridge has five spans, each with a length of 6.4 m. The clearance above the water level is 5.0 m. The superstructure was a 450 mm-thick continuous RC slab, and the substructure was capping beams and 1 m-diameter RC double-column piers. A photo of the bridge is shown in Figure 1, including numbering of the bridge piers.

Before the test, a thorough inspection of the bridge was carried out. Significant steel corrosion in most pier columns was detected by visual inspection. The steel corrosion in the bridge decks was also detected by a half-cell potential method, but corrosion degrees were not determined. By using a rebound method, the measured concrete strengths in piers and decks were 39.4 and 55.3 MPa, respectively. Regular reinforcing steel for steel bars in the RC structures was used in the bridge. For bridge piers, the average thickness of concrete covers for transverse and longitudinal reinforcements was 51.6 and 74.2 mm, respectively. For bridge decks, the average cover thickness for transverse and longitudinal reinforcements was 21.2 and 32.6 mm, respectively. By using phenolphthalein as an indicator, the measured carbonation depths for the pier and deck were 1.0 and 2.5 mm, respectively, which were well within the range of the concrete covers.

The bridge is exposed to a typical marine environment and detailed environmental conditions are shown in Table 1. The seawater chlorinity and atmospheric chloride content were measured at a site close to the bridge [34,35]. Other environmental parameters in Table 1 were averaged between the years of 2000 and 2015, using data from the Meteorological Bureau of Shenzhen Municipality. In such surrounding environment, chloride was generally considered to be an important source of steel corrosion for the RC bridge.

### 2.2. Concrete and Steel Samples

Concrete and steel samples were obtained from the bridge to investigate chloride-induced steel corrosion. Two different bridge zones were considered: the atmospheric zone and the tidal zone. The bridge deck was considered for the atmospheric zone. Pier columns within a range of altitudes were subjected to periodic wetting and drying and were considered to be in the tidal zone.

**Figure 2:**
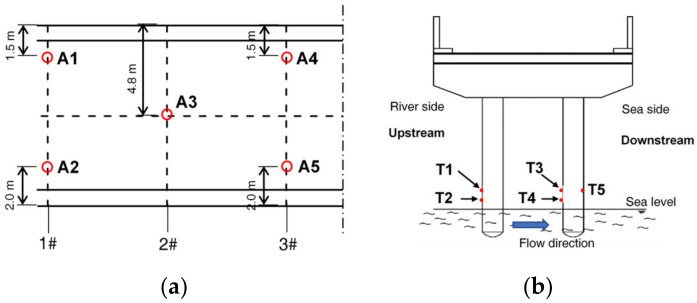
Locations of concrete samples: (**a**) Bridge deck; (**b**) Pier #1.

Ten concrete samples were cored out, with a diameter of 70 mm and a height of 50 mm. As shown in Figure 2a, five concrete samples (A1–A5) were cored out along the western half of the bridge deck, representing the concrete exposed to the atmospheric environment. Four of them were close to the deck side and the other was in the middle of the deck half. Another five concrete samples (T1–T5) were cored out in the tidal zone on the columns of pier #1, as shown in Figure 2b. Pier #1 was selected for testing due to its visible degradation as well as the convenience for sampling. The effect of different altitudes on the column was considered. The samples T1, T3, and T5 were 500 mm above the lowest sea level, and samples T2 and T4 were 100 mm above the lowest sea level. Samples T1–T4 were on the front faces of the column to the water flow, and sample T5 was on the back face. Steel samples were cut out close to A1 and T1 for the tests of steel corrosion in the atmospheric and tidal zones, respectively. The steel samples had a diameter of 20 mm and a length of 250 mm.

### 2.3. Measurement of Chloride Ion Content

Concrete samples A1–A5 and T1–T5 were used to investigate the chloride distribution within the concrete in the atmospheric and tidal zones. Cylindrical concrete samples were ground to powder from the exposed surface to a depth of 16 mm at 1 mm intervals. From 16 to 30 mm, the samples were ground at an interval of 2 mm. The powders were oven-dried at 80 ± 5 °C for 6–8 h and cooled to room temperature in a desiccator.

According to the AASHTO T260-297 standard [36], the free chloride content of the concrete samples was measured by water-soluble extraction. Potentiometric titration was conducted by using a 0.01 mol/L silver nitrate (AgNO_3_) solution. The consumed volume of AgNO_3_ solution was recorded by an automatic potentiometer titrator (version: 809 Titrando, Metrohm, Herisau, Switzerland) and was used to calculate the chloride content. The chloride content was expressed as the mass percentage of chloride ion in concrete. The chloride profile was obtained by plotting the chloride content against the corresponding depth from the exposed surface.

### 2.4. Microscopy Tests

The microstructure of concrete in different environmental zones can be obtained by using MIP and SEM techniques [37]. MIP was used to detect the porosity and pore size distribution of concrete in the bridge. In the test, the volume of intruded mercury into the material was measured at different pressure levels. According to Washburn’s equation, pores with diameters larger than *d* in the materials are filled with mercury at an applied pressure *P*. The pore diameter d corresponding to the applied pressure *P* is given by [37]

(1) $$d=\frac{-4\gamma \cos\theta }{P}$$

where *γ* is surface tension (480 mN/m) and *θ* is the contact angle between mercury and the pore wall (130°).

Tested specimens for MIP were crushed particles with sizes of 10–15 mm from the concrete samples T1, T4, T5, and A1. The specimens were oven-dried at 60 °C for 24 h and then placed into the MIP instrument (AutoPore IV 9500, Micromeritics, Norcross, GA, USA), which had a minimum detectable pore diameter of 6 nm. The applied pressure was increased stepwise to the highest level and the intruded volume of mercury was recorded at each step.

SEM was used to study the microstructural morphology of concrete in different marine environmental zones by a device, the microscope S-3400N (Hitachi, Tokyo, Japan). The cover concrete from the bridge deck and pier columns was crushed to obtain small samples. The sample surfaces were polished and they were then oven-dried at 60 °C for 24 h. Before the SEM test, the sample was coated with a thin layer of gold to prevent charging. The microstructure of the steel rust from the steel samples was also tested by SEM.

### 2.5. Chemical Phase Analysis

XRD was used to analyze the phase composition of concrete and steel rust by a device (model: D8 ADVANCE, Bruker, Karlsruhe, Germany). The samples A1 and T1 at a depth of 5 mm and the rust layers of the steel samples were ground to powders of sizes between 0.1 and 10 µm before the analysis. Generator settings were 40 kV and 40 mA, and XRD data were collected over a 2*θ* range of 5–80°, with a step width of 0.02° and a counting time of 0.2 s/step.

Raman spectroscopy is a non-destructive technique for characterization of various oxides and oxyhydroxides in steel rust [24,38]. It was also employed in this study to detect the phase composition of the rust in the steel samples. A Raman spectrum is produced by inelastic scattering of light on the material. During the inelastic scattering, the frequency difference between incident light and scattered light gives information about the molecular structure of the material. Using a Raman spectrometer (Renishaw, Gloucestershire, UK), the Raman spectrum of the rust on the surface of the steel rust was obtained.

## 3. Results and Discussion

### 3.1. Concrete Microstructure

**Figure 3:**
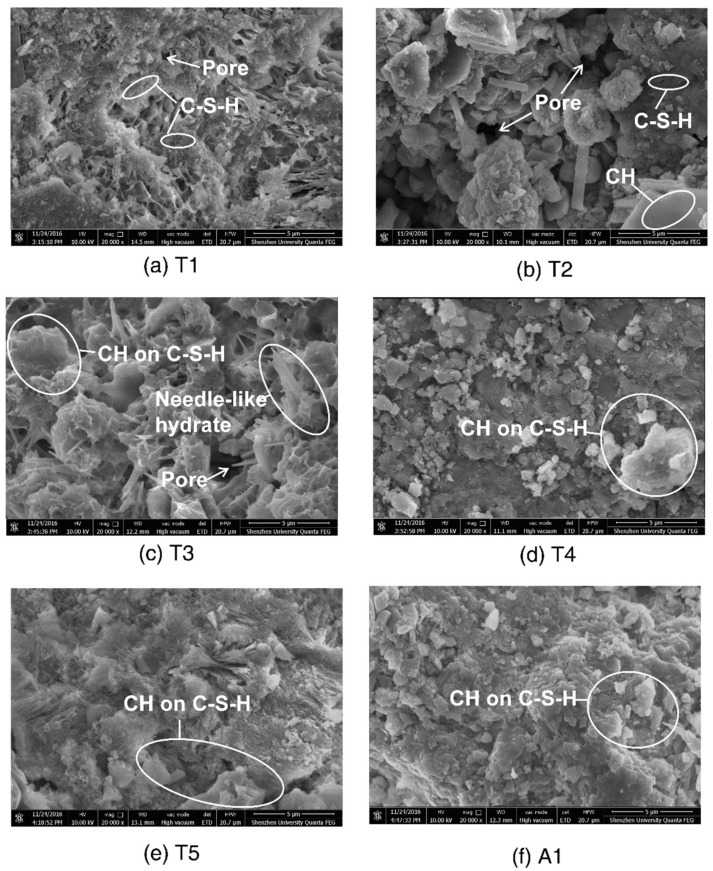
SEM micrographs of concrete samples from the atmospheric and tidal zones (magnification by 20,000 times): (**a**) T1; (**b**) T2; (**c**) T3; (**d**) T4; (**e**) T5; (**f**) A1.

**Figure 4:**
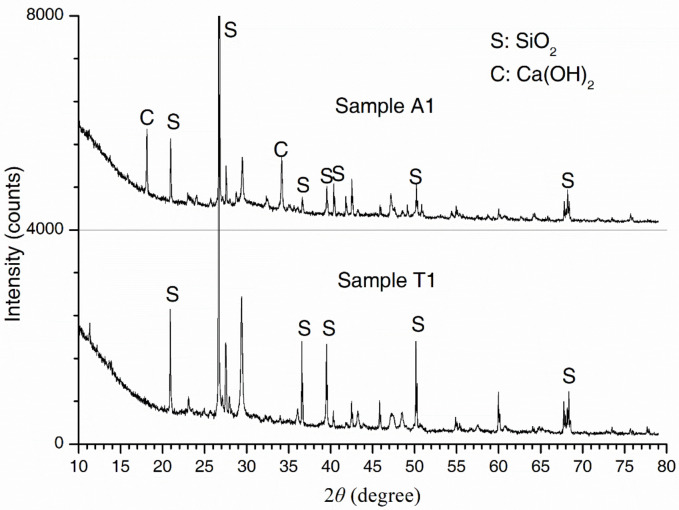
XRD spectra of concrete samples.

**Figure 5:**
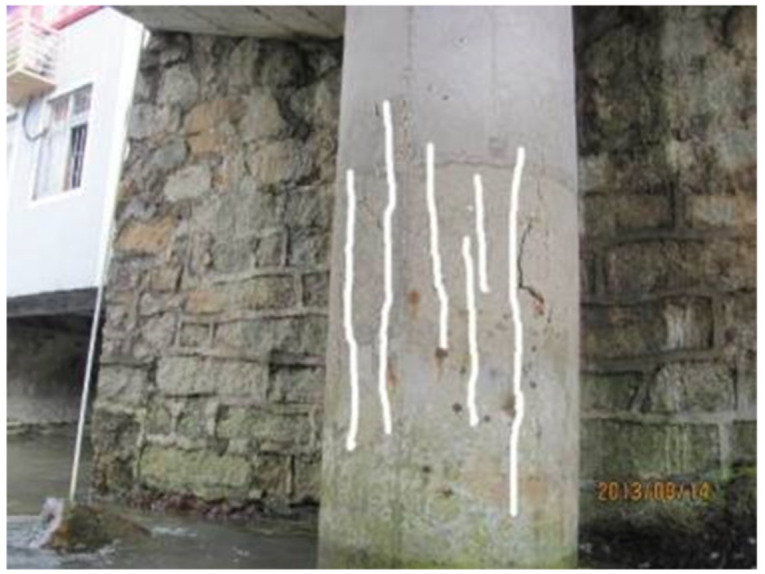
Cracks on the surface of pier #1.

SEM micrographs of the concrete samples from the atmospheric and tidal zones are shown in Figure 3. In typical micrographs, C-S-H, Ca(OH)_2_ (denoted as CH) grains and needle-like ettringite crystals were found. Samples T1–T3 exhibited a looser and more porous structure. By contrast, samples T4, T5, and A1 had more compact structures with well-distributed hydration products. XRD spectra of concrete samples A1 and T1 are shown in Figure 4. The most intense peaks in both spectra were attributed to SiO_2_. CH was detected in A1 but not in T1. This result could possibly be explained by the leaching of solid CH in concrete subjected to flowing river water. CH leaching resulted in more pores and produced more passages for calcium ion diffusion towards the external water, which further promoted leaching [39]. Additionally, cracks were observed on the pier, as shown in Figure 5. The cracking was probably due to mechanical loads and/or expansion of corroded steel bars, as rust stains were seen on the surface of the pier. Water flowed within the cracked concrete more quickly and thus the leaching was accelerated.

**Figure 6:**
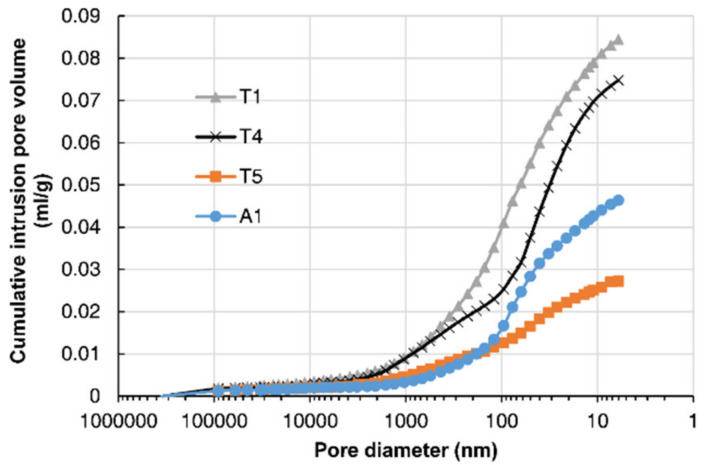
Cumulative intrusion pore volume measured by MIP.

**Figure 7:**
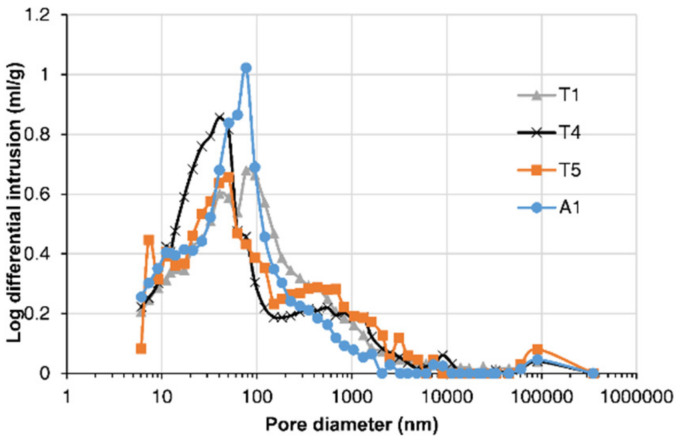
Comparison of measured pore size distributions.

MIP results for concrete samples from the piers and deck of the bridge are shown in Figure 6. The cumulative intrusion volume was plotted against the pore diameter of the concrete. The comparison of the pier’s results showed that the porosities of concrete samples T1 and T4 were significantly larger than the porosity of T5. Concretes T1 and T4 faced upstream and the water impact on them was greater, which caused damage to the cover concrete. Consequently, the effect of the solid CH leaching on increasing porosity was also more significant. It was also found that T1’s porosity was higher than T4′s. The log differential pore size distribution was obtained by dividing the cumulative intrusion volumes at two adjacent sizes by the difference of their log sizes. The pore size distribution in Figure 7 indicated that concrete sample T4 had a larger proportion of small pores than T1, probably due to the effect of chloride binding on the concrete’s microstructure, which will be discussed in the following section.

### 3.2. Chloride Distribution within the Concrete Cover

**Figure 8:**
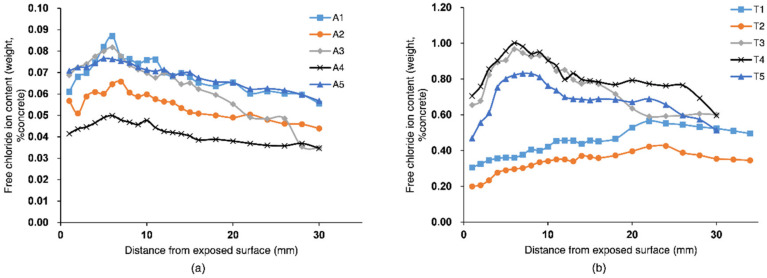
Chloride profiles within the concrete: (**a**) in the atmospheric zone and (**b**) in the tidal zone.

Distributions of chloride ions within the concrete in the atmospheric and tidal zones are shown in Figure 8. Chloride ion content first increased to a peak value and then decreased with the distance from the exposed surface. This was due to the periodic wetting and drying conditions. It was noted that the wetting period for the deck concrete was mainly caused by rainfall. During the wetting period, chloride ions in the external water were brought into the concrete rapidly, along with water absorption, resulting in a peak near the exposed surface. During the drying period, chloride diffused from the high-chloride-content region near the exposed surface to the deeper region with the low chloride content. The external seawater with chloride ions moved into the concrete and chloride diffused into the deeper concrete throughout the wetting-drying cycles.

The depth for the convection zone of the chloride profile is related to the influencing depth for water adsorption. The convection depth was defined as the distance from the exposed surface to the peak of a chloride profile. The results in Figure 8 show that the convection depths for T1 and T2 were 22–24 mm, while other samples’ convection depths were 6–7 mm. Greater convection depths for T1 and T2 were possibly due to cracking on their pier, as shown in Figure 5. This was consistent with Ye et al.’s result [40], who found that the convection depth for sound concrete was 5–15 mm and the convection depth for cracked concrete was greater, which depended on the crack width. The convection depths ranged from 20 to 35 mm when the crack width increased from 0.1 to 0.2 mm. The comparison of concrete samples at different altitudes in Figure 8 showed that the chloride profiles for T3 and T4 were quite similar, while the profile of T1 was noticeably above T2′s. Concrete at different altitudes in the pier column had different drying times in the tidal cycle. During drying periods, due to the cracking of T1 and T2, the drying rates for T1 and T2 were higher than the rates for T3 and T4. Then, more seawater with chloride ions was brought in during the subsequent wetting periods. As the drying time increased with increasing altitude, concrete near the exposed surface for T1 was dried more sufficiently than T2. Consequently, a greater amount of chloride was absorbed for T1 during the subsequent wetting. According to Zhang and Jin’s work [10], as the altitudes further increased, the chloride content in the concrete would decrease, due to the decreased time for the seawater adsorption.

The comparison of chloride profiles in Figure 8 showed that chloride content in the tidal zone was significantly larger than that in the atmospheric zone. This was due to different boundary conditions for the chloride transport. Chloride ion was absorbed from seawater continually under cyclic wetting-drying conditions in the tidal zone, while in the atmospheric zone, surface chloride accumulated from chloride in the atmosphere and then diffused into the concrete. The results also showed that the chloride profiles for T1 and T2 were lower than the profiles for other pier concrete samples. This was because the concrete samples T1 and T2 were closer to the river water, where ambient chloride concentrations were lower. The chloride content for T5 was also lower than T3 and T4′s, even though these concrete samples were located next to each other and had similar boundary chloride concentrations. This could be explained by the lower porosity of T5, as shown in Figure 6. The lower porosity led to a decreased number of pathways for chloride transport within the concrete.

The rate of chloride transport in concrete was influenced by the concrete’s microstructure. Chloride transport in concrete could also in turn influence its microstructure. In Figure 7, the comparison between pore size distributions of T1 and T4 indicated that large pores were filled, leading to a finer pore structure for T4. This was probably due to the influence of chloride binding on the concrete’s microstructure. As shown in Figure 8b, the chloride content for T4 was larger than T1′s, so the effect of binding on decreasing the concrete’s porosity was more significant for T4. Chloride in the concrete was bound by physical adsorption to the solid surface and chemical reactions with C_3_A, C_4_AF, and their hydration products to form Friedel’s salt [41]. Chloride binding could decrease concrete porosity as a result of a filling effect. Yuan et al. [42] demonstrated this effect by comparing MIP results of the concrete after a steady-state chloride migration test to the concrete stored in saturated limestone water.

### 3.3. Evaluated Chloride Diffusivity and Surface Chloride Concentration

It is widely believed that the chloride diffusion in concrete follows Fick’s law. Therefore, the chloride diffusion in a one-dimensional form can be expressed by the following Equation [43]:

(2) $$\frac{\partial C}{\partial t}=D_{c}\frac{\partial^{2}C}{\partial x^{2}}$$

where *C* is the chloride concentration (% of concrete by weight), and *D*_c_ is the chloride diffusivity (m^2^/s).

The initial chloride concentration in the concrete is 0, and the boundary chloride concentration is set as *C*_sa_. Given the initial and boundary conditions for chloride concentration in Equations (3) and (4), the closed-form solution for Equation (2) can be obtained under an assumption of a semi-infinite solid [44]:

(3) $$C\left(x,0\right)=0\begin{array}{cc} & x>0 \end{array}$$

(4) $$C\left(0,t\right)=C_{\mathrm{sa}}\left(t\right)\begin{array}{cc} & t\geq 0 \end{array}$$

(5) $$C\left(x,t\right)=C_{\mathrm{sa}} [e^{\frac{-x^{2}}{4D_{c}t}}-\frac{x\sqrt{\pi }}{2\sqrt{D_{c}t}}erfc\left(\frac{x}{2\sqrt{D_{c}t}}\right)]$$

where *C*(*x*,*t*) is the chloride concentration at distance *x* from the surface at time *t*, *C*_sa_ is the surface chloride concentration, and erfc( ) is the complementary error function.

**Figure 9:**
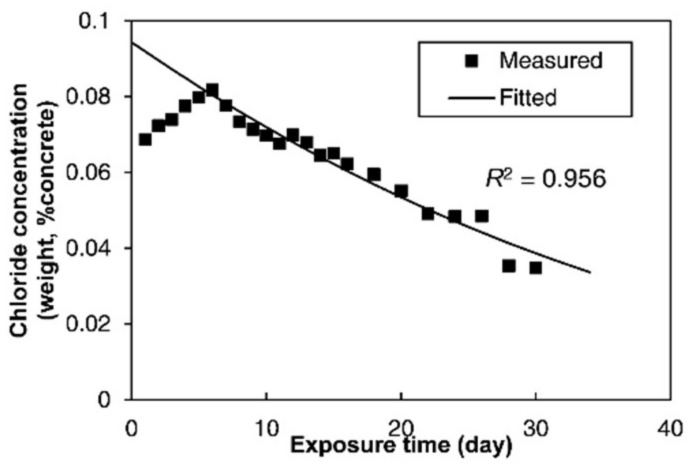
Fitting of the measured chloride profile based on Fick’s equation.

In Equation (5), two key parameters, *C*_sa_ and *D*_c_, determine the evolution of chloride distribution within the concrete. These two parameters were obtained by fitting the diffusion part of the measured chloride profiles at the inner region based on Equation (3), as shown in Figure 9. The fitted chloride diffusivity and surface chloride concentration at different locations of the bridge are presented in Table 2, and the coefficients of determination are also presented. The results showed that the diffusivity of the concrete in the upstream column T1 was the largest, while that at the center of the deck A3 was the smallest. The chloride diffusivity can be related to the pore structure of concrete since the chloride diffuses through the pore networks in the material. The detected pore volume by MIP in Figure 6 indicated that the concrete T1 had the greatest porosity, which was consistent with the result that T1 had the largest chloride diffusivity. The influence of the porosity on the diffusivity was also detected by the field tests under a marine environment carried out by Zhang et al. [45]. However, the concrete T4 had greater porosity than A1 and T5, while the chloride diffusivity of T4 was smaller than that of A1 and T5. This was possibly due to the smallest critical pore size at the peak of the pore size distribution for T4. The results may indicate that both porosity and critical pore size influenced the chloride diffusion rate. It was also found by Yang [46] that the chloride migration coefficients were linearly related to the critical pore diameter.

The estimated surface chloride concentration *C*_sa_ in Table 2 showed that *C*_sa_ was dependent on the exposure condition for the concrete samples. The surface chloride concentration on the bridge deck was significantly larger than that on the column. This is because the surface chloride on the deck was accumulated gradually from the deposition of airborne chloride, while the pier column was exposed to seawater. It was also found that *C*_sa_ for the upstream column close to the river side was smaller than that for the downstream column close to the sea side. The coefficient of variability of the measured *C*_sa_ for the bridge deck was 19.5% and that for the pier column was 21.2%. On the other hand, the coefficient of variability of the measured diffusivity for the bridge deck was 47.2% and that for the pier column was 70.0%. The results implied that the quality of the concrete in the same bridge was not uniform. Additionally, the variability for different structural elements was significantly different, which was also revealed by the results by Salta et al. [47]. The variability of the material parameters for concrete is an essential factor in modeling the service life of the structures.

### 3.4. Steel Corrosion

**Figure 10:**
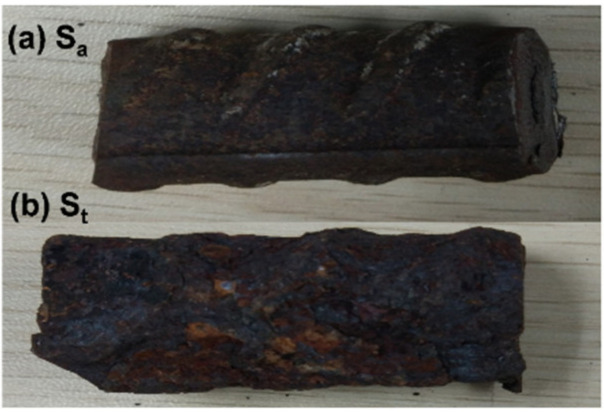
Steel samples in (**a**) the atmospheric zone (Sa) and (**b**) the tidal zone (St).

Chloride transports from the ambient environment into concrete and reaches a steel bar surface. When the chloride content on the steel surface exceeds a critical value, the passive film of the steel is damaged and steel corrosion occurs. The samples of steel bars in the atmospheric zone and the tidal zone (*S*_a_ and *S*_t_, respectively) were obtained from the bridge, as shown in Figure 10. Some rust stains were found on the more intact surface of the sample *S*_a_, while the sample *S*_t_ corroded more severely and its surface was much rougher. On the outer surface of *S*_a_, mostly black areas were seen, with some ochre rust. By contrast, more orange or ochre areas were found on the outer surface of *S*_t_. Differently colored areas possibly imply different rust phases.

**Figure 11:**
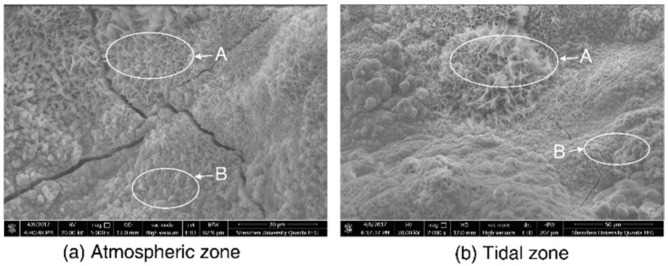
Scanning electron images of steel rust in the (**a**) atmospheric and (**b**) tidal zone.

The corrosion products of steel are composed of various oxides and oxyhydroxides, the formation of which depends on steel composition and environmental conditions. The morphologies of the rust surfaces for *S*_t_ and *S*_a_ were detected by SEM, as shown in Figure 11. The formation “A” was composed of thin plates with sharp edges in a honeycomb-like or net-like pattern, while the formation “B” was composed of dense sandy grains. According to Razvan and Raman’s work [48], formations “A” and “B” corresponded to α-FeOOH and γ-FeOOH, respectively.

**Figure 12:**
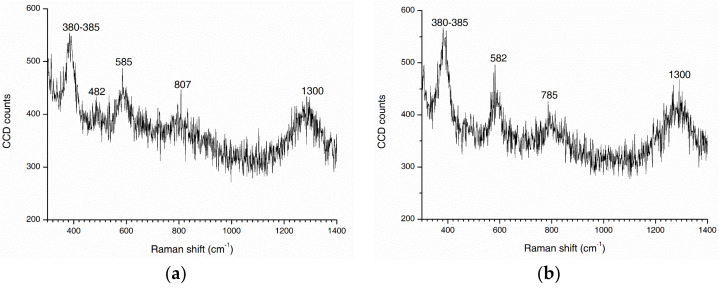
Raman spectra of rust samples in (**a**) the atmospheric zone and (**b**) the tidal zone.

Raman spectra of steel rust in the atmospheric and tidal zones are shown in Figure 12. The peaks of the spectra correspond to specific rust phases. De la Fuente et al. summarized wavelength shift ranges for each rust phase based on different bibliographic sources in a recent paper [49]. Comparison between measured and reported results indicated that the strongest peak at 380–385 cm^−1^ in Figure 12 possibly corresponded to a mixture of α-FeOOH, β-FeOOH, and γ-FeOOH. Peaks at 482 and 1300 cm^−1^ corresponded to α-FeOOH and γ-FeOOH, respectively. However, the intense peaks at 582–585, 785, and 807 cm^−1^ could not be clearly assigned to any rust phase. This may be because of different degrees of crystallinity of the measured steel exposed to the marine environment and the reference samples. The sample from the tidal zone also had a stronger peak at 380–385 cm^−1^ than the sample from the atmospheric zone. This indicated a greater degree of corrosion of the steel in the tidal zone.

**Figure 13:**
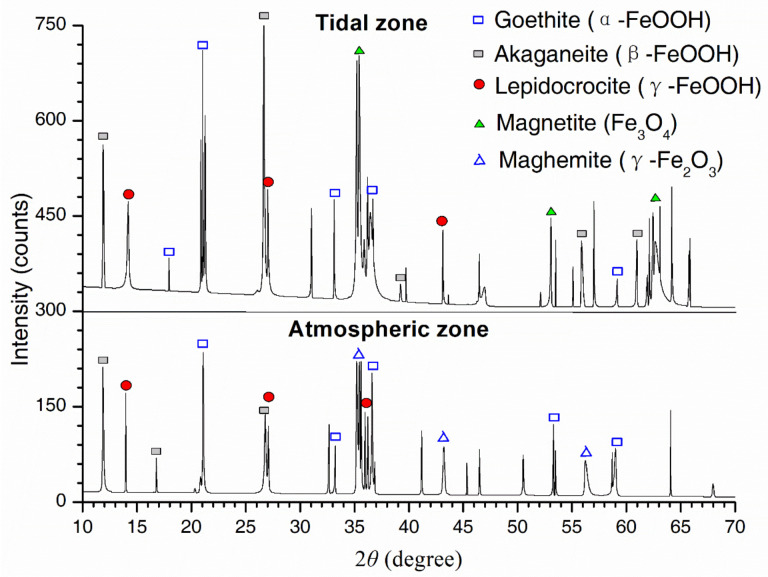
Peak profiles from XRD for rust samples in the atmospheric and tidal zones.

Rust phases of samples were also identified by XRD, as shown in Figure 13. The results also showed that the peak intensity was greater for the tidal zone than for the atmospheric zone, which indicated more severe corrosion in the tidal zone. The steel corrosion was accelerated if exposed to wetting-drying cycles in the tidal zone [50]. During the wetting period, the dissolved oxygen content at the interface between the steel substrate and the solution was very small, because of a thick water layer on the steel surface. As a result, the cathodic reaction led to the reduction of γ-FeOOH. During the drying period, the water layer on the steel surface became thinner and the oxygen supply was sufficient. As a result, the reduced γ-FeOOH was re-oxidized to form γ-FeOOH. Due to the reduction and oxidation of γ-FeOOH during wetting and drying, respectively, the metallic dissolution rate was not limited by the slow diffusion of oxygen.

In the marine environment, high chloride concentration causes the formation of β-FeOOH, which drastically accelerates steel corrosion. Similar to γ-FeOOH, β-FeOOH can be reduced electrochemically by Fe during the wetting period. The reducing capacity of β-FeOOH is even greater than that of γ-FeOOH [50]. The chloride profiles within the concrete cover (Figure 8) indicated that the chloride concentration on the surface of the steel in the tidal zone was larger. As a result, more β-FeOOH was produced and its accelerating effect on corrosion during wetting–dying cycles became more significant. Moreover, more severe concrete cracking was found in the tidal zone, due to the impact of water flow and/or expansion of corrosion products. The expansion coefficient of β-FeOOH is 3.53, which is the highest among the main corrosion products [51]. The cracking of the concrete cover would accelerate chloride diffusion and the periodic wetting-drying of the steel.

**Figure 14:**
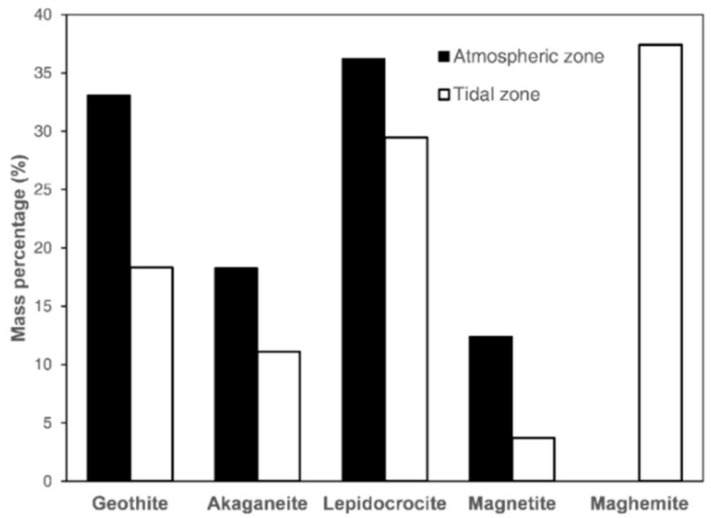
Mass fractions of rust phases by XRD.

Based on the XRD results, a quantitative analysis was conducted by using a reference intensity ration (RIR) method. The results in Figure 14 show that γ-FeOOH and α-FeOOH were detected as major rust phases in the atmospheric zone, while lower proportions of β-FeOOH and Fe_3_O_4_ were detected, and γ-Fe_2_O_3_ was not found. For the rust in the tidal zone, the proportions of α-FeOOH, γ-FeOOH, β-FeOOH, and Fe_3_O_4_ decreased, while the proportion of γ-Fe_2_O_3_ significantly increased and became the major phase. This implied that Fe_3_O_4_ was transformed to γ-Fe_2_O_3_ only when steel corrosion exceeded a certain extent. Similarly, the investigation of a RC beam exposed to a marine environment by Poupard et al. [25] also indicated that rust was only composed of Fe_3_O_4_ in low-corroded regions, while rust was mainly composed of α-FeOOH and γ-Fe_2_O_3_ in high-corroded regions. These results could possibly be explained by cracking of rust layers. Fe_3_O_4_ was formed at the steel substrate–rust interface, where a consolidated rust layer was covered and oxygen was initially depleted. With the progress of steel corrosion, the rust layer cracked more extensively or even was exfoliated, so oxygen could possibly reach the steel substrate and react with Fe_3_O_4_ to form γ-Fe_2_O_3_.

## 4. Conclusions

In this paper, the chloride distribution within concrete covers and the chloride-induced steel corrosion in a 30-year-old RC bridge exposed to a marine environment were investigated. This study enhances the understanding of chloride-induced steel corrosion under a natural condition. Based on experimental results, the following conclusions can be drawn:

1. The porosity of concrete in a pier column facing upstream was greater due to the water impact and CH leaching. Concrete with a higher chloride content had lower porosity and a larger proportion of small pores.
2. The convection zone depths of the chloride profiles were all in the range of 6–7 mm, except that the convection depths for the cracked concrete were 22–24 mm.
3. The coefficients of variability of evaluated concrete chloride diffusivity for the bridge deck and the pier column were significantly different.
4. The rust phases were primarily lepidocrocite and goethite in the atmospheric zone and lepidocrocite and maghemite in the tidal zone. This may imply that transformation of magnetite to maghemite occurred for a greater degree of corrosion.

## Figures and Tables

**Table 1:** Environmental conditions.

Condition	Value
Seawater chlorinity	12.93‰
Chloride content in atmosphere	0.05–0.12 mg/m^3^
Temperature	Annual average: 22.5 °C Range: 8.7–36.8 °C
Annual average relative humidity	77% Range: 55–80%
Annual average rainfall	1846 mm Range: 913–2662 mm
Prevailing wind direction	Southeast (SE)
Annual average wind speed	3.0 m/s Range: 2.5–3.3 m/s

**Table 2:** Estimated chloride diffusivity and surface chloride concentration.

Specimen Number	Chloride Diffusivity (×10^−12^ m^2^/s)	Surface Chloride Concentration (%)	Coefficients of Determination *R*^2^
A1	4.15	0.086	0.880
A2	4.10	0.068	0.904
A3	1.24	0.094	0.956
A4	4.26	0.052	0.895
A5	7.59	0.080	0.978
T1	8.93	0.706	0.990
T2	2.69	0.650	0.866
T3	1.77	1.100	0.939
T4	1.81	1.094	0.904
T5	3.82	0.892	0.848
